# Effect of Ranirestat, a New Aldose Reductase Inhibitor, on Diabetic Retinopathy in SDT Rats

**DOI:** 10.1155/2014/672590

**Published:** 2014-08-25

**Authors:** Fumihiko Toyoda, Yoshiaki Tanaka, Ayumi Ota, Machiko Shimmura, Nozomi Kinoshita, Hiroko Takano, Takafumi Matsumoto, Junichi Tsuji, Akihiro Kakehashi

**Affiliations:** ^1^Department of Ophthalmology, Saitama Medical Center, Jichi Medical University, 1-847 Amanuma-cho, Omiya-ku, Saitama 330-8503, Japan; ^2^Drug Development Research Laboratories, Sumitomo Dainippon Pharma Co., Ltd., 6-8-2 Doshomachi, Chuo-ku, Osaka 541-0045, Japan

## Abstract

*Purpose*. To evaluate the effect of ranirestat, a new aldose reductase inhibitor (ARI), on diabetic retinopathy (DR) in Spontaneously Diabetic Torii (SDT) rats. *Methods*. The animals were divided into six groups, normal Sprague-Dawley rats (*n* = 8), untreated SDT rats (*n* = 9), ranirestat-treated SDT rats (0.1, 1.0, and 10 mg/kg/day, *n* = 7, 8, and 6, resp.), and epalrestat-treated SDT rats (100 mg/kg/day, *n* = 7). Treated rats received oral ranirestat or epalrestat once daily for 40 weeks after the onset of diabetes. After the eyes were enucleated, the retinal thickness and the area of stained glial fibrillary acidic protein (GFAP) were measured. *Results*. The retinas in the untreated group were significantly thicker than those in the normal and ranirestat-treated (0.1, 1.0, and 10 mg/kg/day) groups. The immunostained area of GFAP in the untreated group was significantly larger than that in the normal and ranirestat-treated (1.0 and 10 mg/kg/day) groups. There were no significant differences between the untreated group and epalrestat-treated group in the retinal thickness and the area of stained GFAP. *Conclusion*. Ranirestat reduced the retinal thickness and the area of stained GFAP in SDT rats and might suppress DR and have a neuroprotective effect on diabetic retinas.

## 1. Introduction

Diabetic retinopathy (DR) is a leading cause of visual loss and blindness in adults in most developed countries [1]. Appropriate and effective treatment against DR needs to be developed. Surgical treatments for DR, such as laser photocoagulation and vitrectomy, are well developed. The effect of laser photocoagulation for preventing and treating proliferative diabetic retinopathy (PDR) and diabetic macular edema (DME) has been proved [2, 3]. However, the effect of laser photocoagulation for treating PDR is somewhat limited and cannot be performed in patients with opaque media. The effect of grid laser photocoagulation for treating DME also is limited and advanced atrophic creep around the macula resulting in severe visual impairment has developed in some patients with DME treated with this therapy [4]. Vitrectomy, an established surgical treatment for PDR, is gaining in popularity for DME. However, the visual prognoses of vitrectomy for treating PDR and DME are unsatisfactory. Glycemic control is the primary medical treatment for DR. Several clinical trials have reported that intensive glycemic control reduces the incidence and progression of DR [5–7].

Although glycemic control seems to be the most important approach, achieving acceptable glucose homeostasis is difficult, even in patients who adhere strictly to treatment. Therefore, it is important to find medical options other than glycemic control to prevent DR. The metabolic changes that accompany hyperglycemia, such as activation of the polyol pathway [8], activation of protein kinase C (PKC) [9], increased oxidative stress [10], leukocyte adhesion to the endothelial cells [11], and accumulation of advanced glycation end products (AGEs) [12], are related to the development and progression of diabetic ocular complications. In particular, the polyol pathway is correlated strongly with oxidative stress, activation of PKC, and accumulation of AGEs that lead to induction of vascular endothelial growth factor (VEGF). Intravitreous injections of triamcinolone [13, 14] and anti-VEGF agents [15–17] are recently developed major treatments for DME. However, they are associated with risk of infection due to multiple intravitreous injections and high cost; in addition, they are not indicated for PDR and only for limited cases without pathological changes such as vitreomacular traction.

Among the targeted metabolic factors, we focused on the polyol pathway. A key enzyme in the polyol pathway is aldose reductase (AR), which is found in the retina, lens, and Schwann cells of the peripheral nerves [18]. Our previous study showed an inhibitory effect of fidarestat (SNK-860, Sanwa Kagaku Kenkyusho, Nagoya, Japan), an AR inhibitor (ARI), on the development of DR in SDT rats [19]. Fidarestat suppressed the VEGF levels in the ocular fluid and prevented extensive fluorescein leakage around the optic disc in that study. We also confirmed the effect of a new ARI, ranirestat (AS3201, Sumitomo Dainippon Pharmaceutical Co., Osaka, Japan), on DR in SDT rats [20]. Ranirestat suppressed accumulation of VEGF and N*ε*-(carboxymethyl) lysine in the retina of SDT rats in that study. We confirmed that ranirestat also suppressed diabetic cataract and neuropathy in SDT rats [21]. However, we did not confirm whether ranirestat prevents retinal edema and neurodegeneration in diabetic retinas. In the current study, we evaluated the effect of ranirestat on the development of DR by preventing retinal edema and on the neurodegeneration in diabetic retinas by preventing glial fibrillary acidic protein (GFAP) accumulation within the retina in SDT rats.

## 2. Materials and Methods

### 2.1. Animals

The care and handling of animals were in accordance with the Association for Research in Vision and Ophthalmology Statement for the Use of Animals in Ophthalmic and Visual Research and the Jichi Medical University Animal Care and Use Committee. Some procedures used in this study were the same as the methods we reported previously, and the animals used in this study were the same as those we used in a previous study [21]. We obtained male SDT rats and SD rats from CLEA, Inc. (Tokyo, Japan). All SDT rats were confirmed to be diabetic based on a nonfasting blood glucose concentration exceeding 350 mg/dL. The SDT rats were diagnosed with diabetes by 12 to 20 weeks after birth. All rats were fed standard rat chow (CRF-1, Oriental Yeast, Inc., Tokyo, Japan). Treated rats received oral ranirestat or epalrestat, an ARI obtained from Sumitomo Dainippon Pharmaceutical Co. (Osaka, Japan), which served as a positive control, once daily for 40 weeks after the onset of diabetes. Untreated rats and normal SD rats received no drug for 40 weeks. The animals were divided into six groups: normal SD rats (*n* = 8), untreated SDT rats (*n* = 9), ranirestat-treated (0.1 mg/kg/day for 40 weeks) SDT rats (*n* = 7), ranirestat-treated (1.0 mg/kg/day for 40 weeks) SDT rats (*n* = 8), ranirestat-treated (10 mg/kg/day for 40 weeks) SDT rats (*n* = 6), and epalrestat-treated (100 mg/kg/day for 40 weeks) SDT rats (*n* = 7). All rats were over 50 weeks old.

The untreated SDT rats that were 31 weeks old and normal SD rats that were 33 weeks old were dissected to examine the developmental process (*n* = 4,4), and we compared these rats with over 50-week-old normal SD rats and untreated SDT rats.

### 2.2. Measurement of Body Weight, Blood Glucose, and Glycated Hemoglobin

Body weight, blood glucose, and glycated hemoglobin (HbA1c) were measured once monthly. Blood samples were collected from the tail vein of nonfasting rats to measure the blood glucose and HbA1c. Blood glucose was measured by the hexokinase-G-6-PDH method (L type Wako Glu2, Wako Pure Chemical Industries, Ltd., Osaka, Japan). HbA1c was measured using an automated glycohemoglobin analyzer (HLC-723GHb V, Tosoh Corporation, Tokyo, Japan) [21].

### 2.3. Ocular Histopathology

Some ocular histopathology procedures were the same as the methods we reported previously [21]. Under deep anesthesia induced by an intraperitoneal injection of pentobarbital sodium (25 mg/kg body weight, Nembutal, Sumitomo Dainippon Pharmaceutical Co., Ltd., Osaka, Japan), the eyes were enucleated for conventional histopathologic studies and placed in a fixative (Super Fix KY-500, Kurabo, Japan). The fixed eyes were washed in 0.1% mol/L cacodylate buffer and embedded in paraffin. The paraffin block was sectioned to 4 *μ*m and stained with hematoxylin and eosin for conventional histopathologic examination. The immunohistochemical procedures were based on the standard avidin-biotin horseradish peroxidase method using each antibody and developed with AEC Substrate-Chromogen (Dakocytomation, Carpinteria, CA, USA). GFAP mouse monoclonal antibody (Cell Signaling Technology, Inc., Danvers, MA, USA) was used at a dilution of 1 : 50. Bovine serum was used as a primary antibody for negative control of the immunostaining.

### 2.4. Measurement of Retinal Thickness and Area of Stained GFAP

The paraffin blocks sectioned to 4 *μ*m were examined using a polarizing microscope (Olympus BX-51, Olympus Corporation, Tokyo, Japan), and the images were recorded and downloaded using the attached digital camera and software (Olympus DP 72, DP2-BSW, Olympus Corporation). Retinal tissue 300 to 600 *μ*m from the optic disc was observed for retinal changes using the DP2-BSW. The retinal thickness and the area of stained GFAP 300 *μ*m in width were measured using ImageJ software (National Institutes of Health, Bethesda, MD, USA). The retinal thickness was measured as the distance between the retinal internal limiting membrane (ILM) and the retinal pigment epithelium.

### 2.5. Statistical Analysis

All values were expressed as the mean ± standard deviation. The Mann-Whitney *U* test and Steel's test were used for comparisons between each group. Excel Tokei 2006 software (Social Survey Research Information Co., Ltd., Tokyo, Japan) was used for statistical analysis. *P* < 0.05 was considered statistically significant.

## 3. Results

### 3.1. Body Weight, Blood Glucose, and Glycated Hemoglobin

**Figure 1:**
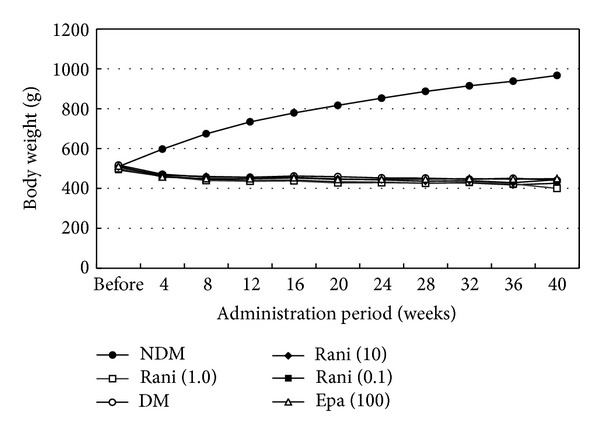
Body weight of the study animals. The SD rats are heavier than the SDT rats with or without treatment. NDM: normal SD rats; DM: untreated SDT rats; rani (0.1): ranirestat-treated SDT rats (0.1 mg/kg/day); rani (1.0): ranirestat-treated SDT rats (1.0 mg/kg/day); rani (10): ranirestat-treated SDT rats (10 mg/kg/day); epa (100): epalrestat-treated SDT rats (100 mg/kg/day) [21].

**Figure 2:**
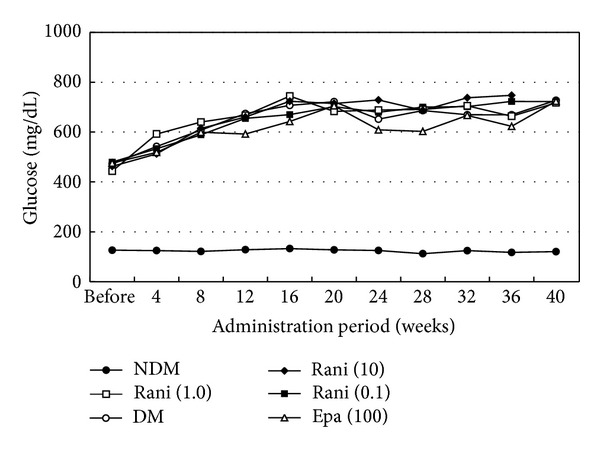
Blood glucose levels of the study animals. The mean blood glucose levels of the SD rats are significantly lower than those of the SDT rats with or without treatment. There is no significant difference in the blood levels among the SDT rats with or without treatment. NDM: normal SD rats; DM: untreated SDT rats; rani (0.1): ranirestat-treated SDT rats (0.1 mg/kg/day); rani (1.0): ranirestat-treated SDT rats (1.0 mg/kg/day); rani (10): ranirestat-treated SDT rats (10 mg/kg/day); epa (100): epalrestat-treated SDT rats (100 mg/kg/day) [21].

**Figure 3:**
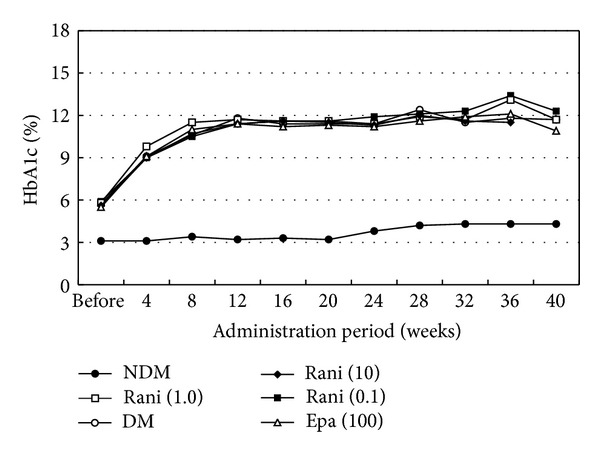
HbA1c levels of the study animals. The mean HbA1c levels of the SD rats are significantly lower than those of the SDT rats with or without treatment. There is no significant difference in the HbA1c levels among the SDT rats with or without treatment. NDM: normal SD rats; DM: untreated SDT rats; rani (0.1): ranirestat-treated SDT rats (0.1 mg/kg/day); rani (1.0): ranirestat-treated SDT rats (1.0 mg/kg/day); rani (10): ranirestat-treated SDT rats (10 mg/kg/day); epa (100): epalrestat-treated SDT rats (100 mg/kg/day) [21].

Figures 1, 2, and 3 show the changes in weight, blood glucose, and HbA1c, respectively, during the study. Compared with the SD rats, the SDT rats were significantly (*P* < 0.01) lighter with and without ARI treatment. The mean blood glucose levels and HbA1c levels of the SDT rats were significantly (*P* < 0.01) higher than those of the SD rats. However, there were no significant differences in the blood glucose levels and HbA1c levels in the treated and untreated rats. Because the ARIs did not affect glycemic control, we did not consider the glycemic effect in this study [21].

### 3.2. Retinal Thickness and Area of Stained GFAP

The values are shown in Table 1. The retinas in the untreated SDT rats were significantly (*P* = 0.0076, *P* = 0.017, *P* = 0.0052, and *P* = 0.016, resp., Steel's test) thicker than those in the normal SD rats and ranirestat-treated (0.1, 1.0, and 10 mg/kg/day) SDT rats. There was no significant (*P* = 0.057, Steel's test) difference between the untreated SDT rats and the epalrestat-treated SDT rats. The stained area in the untreated SDT rats was significantly (*P* = 0.0044, *P* = 0.0052, and *P* = 0.028, resp., Steel's test) larger than that in the normal SD rats and ranirestat-treated (1.0 and 10 mg/kg/day) SDT rats. There was no significant (*P* = 0.11, Steel's test) difference between the untreated SDT rats and the epalrestat-treated SDT rats.

The retinas in the 33-week-old SD rats were significantly (*P* = 0.023, Mann-Whitney *U* test) thicker than those in the SD rats that were over 50 weeks old. The stained area in the SD rats over 50 weeks old was significantly (*P* = 0.0055, Mann-Whitney *U* test) larger than that in the 33-week-old SD rats. The stained area in the untreated SDT rats over 50 weeks old was significantly (*P* = 0.0055, Mann-Whitney *U* test) larger than that in the 31-week-old SDT rats, but there was no significant (*P* = 0.22, Mann-Whitney *U* test) difference in the retinal thickness. The retinas in the 31-week-old SDT rats were significantly (*P* = 0.021, Mann-Whitney *U* test) thicker than those in the 33-week-old SD rats, but there was no significant (*P* = 0.24, Mann-Whitney *U* test) difference in the stained area.

**Figure 4:**
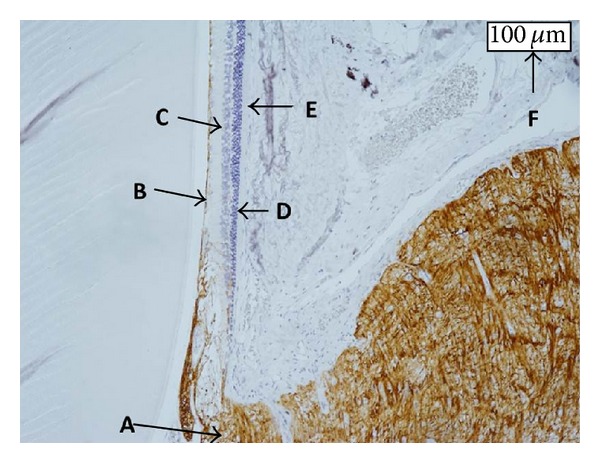
The retina in a normal SD rat older than 50 weeks. The brown region shows stained GFAP. A: optic nerve disc; B: ILM; C: INL; D: outer nuclear layer; E: retinal pigment epithelium; F: index of 100 *μ*m. The area of stained GFAP is limited around the ILM and does not appear around the INL.

**Figure 5:**
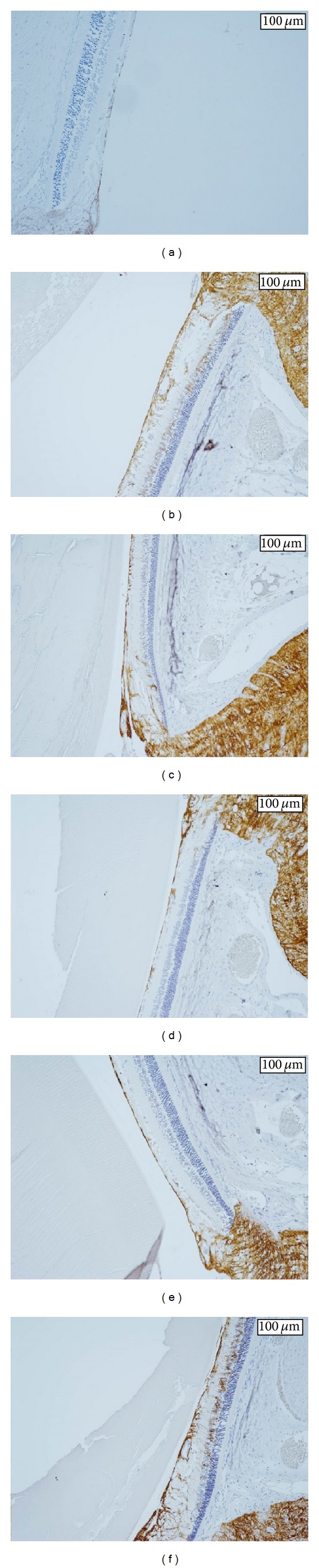
(a) The retina in a 31-week-old untreated SDT rat. The area of stained GFAP is significantly smaller in 31-week-old untreated SDT rats than in untreated SDT rats older than 50 weeks, but there is no significant difference in retinal thickness. (b) The retina in an untreated SDT rat older than 50 weeks. The retina is thicker and the area of stained GFAP is larger compared with the normal SD rat. GFAP is strongly stained from the ILM to around the INL in the untreated SDT rats. (c) The retina in a ranirestat-treated SDT rat (0.1 mg/kg/day). The retina is thinner and the area of stained GFAP is smaller compared with the untreated SDT rat. The stained area, which appears between the ILM and around the INL in the untreated SDT rats, is suppressed. (d) The retina in a ranirestat-treated SDT rat (1.0 mg/kg/day). The effect of ranirestat (1.0 mg/kg/day) is stronger than ranirestat (0.1 mg/kg/day) on the retinal thickness and the area of stained GFAP. (e) The retina in a ranirestat-treated SDT rat (10 mg/kg/day). Although the area of stained GFAP in the ranirestat-treated SDT rat (10 mg/kg/day) is suppressed compared with the ranirestat-treated SDT rat (0.1 mg/kg/day), the difference in retinal thickness is not clear. (f) The retina in an epalrestat-treated SDT rat. Epalrestat does not suppress the retinal thickness or the area of stained GFAP. The area of GFAP is intensely stained between the ILM and around the INL.

**Figure 6:**
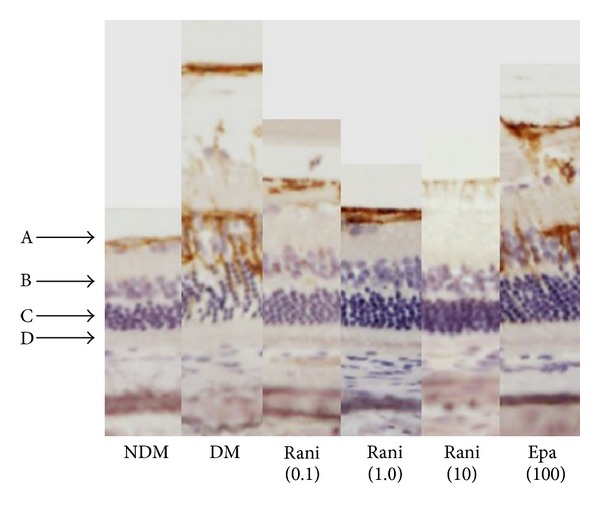
Comparison of retinas in each group. A: ILM; B: INL; C: outer nuclear layer; D: retinal pigment epithelium. NDM: normal SD rats; DM: untreated SDT rats; rani (0.1): ranirestat-treated SDT rats (0.1 mg/kg/day); rani (1.0): ranirestat-treated SDT rats (1.0 mg/kg/day); rani (10): ranirestat-treated SDT rats (10 mg/kg/day); epa (100): epalrestat-treated SDT rats (100 mg/kg/day).

Figures 4, 5, and 6 show the retinas in each group. In the normal SD rats, the region of stained GFAP was minimal near the ILM but became more extensive around the inner nuclear layer (INL) in the untreated SDT rats. The retinas in the SDT rats began to thicken at 33 weeks of age, but the stained area of GFAP had not begun to spread at that time point.

## 4. Discussion

The retinas and the stained area of GFAP in the 33-week-old SD rats were significantly thicker and smaller than those in the SD rats older than 50 weeks in this study. This suggested that the retinas may become thinner and the GFAP in the retina may increase in SD rats over time. The stained area of GFAP in the untreated SDT rats older than 50 weeks was significantly larger than that in the 31-week-old SDT rats, but there was no significant difference in the retinal thickness in this study. This suggested that the increased GFAP in diabetic retinas may begin after retinal edema develops.

We investigated whether ranirestat reduces the retinal thickness in SDT rats. We reported previously that ranirestat suppressed accumulation of VEGF and prevented extensive fluorescein leakage around the optic disc in the retinas of SDT rats [20]. Together with the current results, the results suggested that ranirestat may suppress vascular permeability and prevent DME.

GFAP is a specific component of the glial filaments present in astrocytes [22]. In the central nervous system of higher vertebrates, after injury from trauma, disease, genetic disorders, or chemical insult, astrocytes become reactive and respond in a typical manner, that is, astrogliosis, which is characterized by rapid synthesis of GFAP and increased protein content or immunostaining with the GFAP antibody [23]. It was reported that GFAP also increased locally in some ophthalmic diseases. Yang et al. reported that the immunoreactivity of GFAP was detected and increased in the optic nerve region of a glaucoma model rat [24]. Rungger-Brändle et al. reported that the density of Müller cells and microglia increased and GFAP expression in the Müller cells was prominent in the retinas of a diabetic rat model [25]. Thus, the increase in neuroglial cells and GFAP expression are thought to be associated with onset and development of neuropathy in the diabetic retina. Since neurodegeneration is an early event in the pathogenesis of DR [26], developing treatment for the early disease stage is important.

The effects of diabetes may appear in the retina before funduscopic or morphologic changes occur. Some studies have reported electroretinographic abnormalities. Shirao and Kawasaki [27] reported that the peak latency of the first oscillatory potential (OP) peak was prolonged in early diabetes with no funduscopic abnormality and increased as DR progressed and also that the summed amplitude of the OPs decreased as DR progressed. However, those investigators reported that visual functional disorders were unclear in patients with OP abnormalities who have no or minimal fundus changes and presumed that amacrine cells were the most feasible candidates for OP generation [27]. Aung et al. reported abnormalities in visual acuity, and contrast sensitivity tested using the OptoMotry system (Cerebral-Mechanics, Lethbridge, AB, Canada), and delayed responses specifically in OP implicit times in streptozotocin-induced diabetes mellitus rats before expected onset of diabetes-associated retinal vascular lesions [28]. Previous studies have reported the depth profile of the OPs within the retina. Brindley reported that the maximal amplitude of the oscillations in the frog retina was in the INL [29], and Ogden and Wylie reported that the maximal amplitudes of the first three OPs in the pigeon, chicken, and monkey were at the level of the inner plexiform layer [30, 31]. In the current study, GFAP was strongly stained from the ILM to around the INL in the untreated SDT rats and ranirestat significantly suppressed the area of stained GFAP in the groups treated with 1.0 and 10 mg/kg/day. Considering that together with previous studies of OPs in diabetic retina, neural damage in a diabetic retina may begin in an inner retinal layer; however, GFAP expression in a diabetic retina may begin after the retinal edema develops. The current results suggested that ranirestat may have a neuroprotective effect on diabetic retina, but it may be affected by controlling retinal edema.

Although the mean retinal thickness and the mean area of stained GFAP in epalrestat-treated SDT rats were smaller than those in the untreated SDT rats, epalrestat did not significantly suppress them in the current study. Because the study included a small number of rats, we could not determine whether or not epalrestat was effective for treating DR. The values in the 1.0 mg/kg/day ranirestat group were smaller than those in the 10 mg/kg/day, and the dose-reaction relationship was unclear. The findings also may have resulted from the small number of rats; a larger study with more rats is needed to confirm that effectiveness of epalrestat and the dose-reaction relationship of ranirestat. However, ranirestat may prevent DME and have a neuroprotective effect on diabetic retinas. Further studies of ranirestat should be undertaken.

## Figures and Tables

**Table 1:** The mean retinal thickness and mean area of stained GFAP in each group. 31 wDM: 31-week-old SDT rats; 33 wNDM: 33-week-old normal SD rats; NDM: normal SD rats older than 50 weeks; DM: untreated SD rats older than 50 weeks; rani (0.1): ranirestat-treated SDT rats (0.1 mg/kg/day); rani (1.0): ranirestat-treated SDT rats (1.0 mg/kg/day); rani (10): ranirestat-treated SDT rats (10 mg/kg/day); epa (100): epalrestat-treated SDT rats (100 mg/kg/day).

	31 wDM	33 wNDM	NDM	DM	rani (0.1)	rani (1.0)	rani (10)	epa (100)
Mean retinal thickness (*μ*m)	152.6 ± 12.8	127.2 ± 11.1	90.3 ± 19.8	158.2 ± 23.0	114.7 ± 22.8	93.5 ± 18.2	102.9 ± 17.9	129.7 ± 21.8

*P* value	0.22 (compared with DM) 0.021 (compared with 33 wNDM)	0.023 (compared with NDM)	0.0076 (compared with DM)		0.017 (compared with DM)	0.0052 (compared with DM)	0.016 (compared with DM)	0.057 (compared with DM)

Mean area of stained GFAP (*µ*m^2^)	225.8 ± 132.9	128.0 ± 60.1	1069.8 ± 311.7	2512.5 ± 971.9	1426.8 ± 1131.7	669.0 ± 524.2	789.0 ± 799.0	1671.4 ± 604.6

*P* value	0.0055 (compared with DM) 0.24 (compared with 33 wNDM)	0.0055 (compared with NDM)	0.0044 (compared with DM)		0.13 (compared with DM)	0.0052 (compared with DM)	0.028 (compared with DM)	0.11 (compared with DM)
